# Metabolic-Inflammatory Syndrome: Decoding the Metabolic-Immune Axis for Precision Intervention

**DOI:** 10.14336/AD.2025.1125

**Published:** 2026-10-12

**Authors:** Simin Li, Yawei Shi, Chongge You

**Affiliations:** ^1^Laboratory Medicine Center, The Second Hospital & Clinical Medical School, Lanzhou University, Lanzhou 730030, Gansu, China.; ^2^Department of Pathology, The Second Hospital & Clinical Medical School, Lanzhou University, Lanzhou 730030, Gansu, China

**Keywords:** Metabolic Inflammatory Syndrome, Metabolic Reprogramming, Inflammatory Stimuli, Gut Microbiota

## Abstract

Metabolic-inflammatory syndrome represents a major global health challenge, closely linked to diabetes, cardiovascular disease, obesity, and non-alcoholic fatty liver disease. The core of these disorders involves remodeling of metabolic pathways under nutritional stress and the ensuing chronic inflammatory vicious cycle. This review systematically examines the mechanisms by which metabolic reprogramming drives metabolic-inflammatory syndrome, revealing the significant role of the gut microbiota in this process. Furthermore, it evaluates current metabolic-inflammatory syndrome early-warning systems and proposes innovative therapeutic strategies precisely targeting the metabolic-immune axis, establishing a foundation for enhancing early detection and clinical intervention.

## Introduction

Metabolic-inflammatory syndrome (MIS) represents a global health emergency, accelerated by obesity rates that have tripled since 1975 (www.who.int/mediacentre/factsheets/fs311/en/). This condition drives interconnected epidemics including obesity, cardiovascular disease (CVD), type 2 diabetes mellitus (T2DM) and non-alcoholic fatty liver disease (NAFLD) [1, 2]. Clinical investigations have shown that the risk of tumors is much higher in patients with MIS than in other populations [3].

MIS is not merely a metabolic dysfunction, but a remodeling of metabolic pathways under nutritional stress and a vicious cycle of chronic inflammation [4, 5]. Within its dysregulated metabolic milieu, marked by hyperglycemia, hyperlipidemia, and hypoxia, key immune cells undergo pro-inflammatory metabolic reprogramming [6, 7]. Macrophages shift toward an M1-phenotype, exhibiting glycolytic hyperactivity and mitochondrial dysfunction, driving tissue inflammation and insulin resistance [8, 9]. Effector T cells (Th1/Th17) similarly amplify glycolysis and damaging cytokine release, while regulatory T cell suppression is impaired. Neutrophils contribute through oxidative stress mechanisms [10]. Collectively, these immunometabolic shifts are governed by dysregulated signaling pathways like mTORC1/HIF-1α and suppressed adenosine monophosphate-activated protein kinase (AMPK) [11, 12].

The gut microbiota regulates MIS through its bioactive metabolites [13, 14]. Microbial-derived compounds such as short-chain fatty acids (SCFAs) and lipopolysaccharide (LPS) function as signaling mediators that directly interface metabolic and immune pathways [15]. SCFAs enhance anti-inflammatory responses and promote regulatory T-cell function, whereas pathogenic LPS activates pro-inflammatory TLR4/NF-κB signaling [16]. This activation drives insulin resistance and disrupts lipid homeostasis. Crucially, dysbiosis-induced shifts in microbial metabolite production amplify inflammatory cascades and contribute to heterogeneous manifestations of MIS [17, 18]. Individual variations in microbial functional profiles further enable quantitative prediction of insulin resistance susceptibility. These insights position the gut microbiota as both a dynamic modulator of pathophysiology and a promising target for precision interventions that extend beyond conventional biomarker approaches.

Current management of MIS remains constrained by delayed diagnosis and ineffective single-target therapies. Consequently, high rates of treatment resistance persist, and healthcare expenditures continue to rise. The proposal of the metabolism-inflammation axis has provided new directions for the treatment of metabolic diseases characterized by chronic inflammation (e.g., anti-inflammatory therapy, intestinal flora modulation, NLRP3 inhibitors, etc.).

### The Presentation and Development of Metabolic Inflammatory Syndrome

MIS is an emerging concept in metabolic medicine and inflammation research in recent years [19] (Fig. 1). It has been proposed and developed based on in-depth studies of metabolic syndrome (MS), particularly the central role of chronic inflammation in metabolic disorders (Table 1).

**Figure 1.**
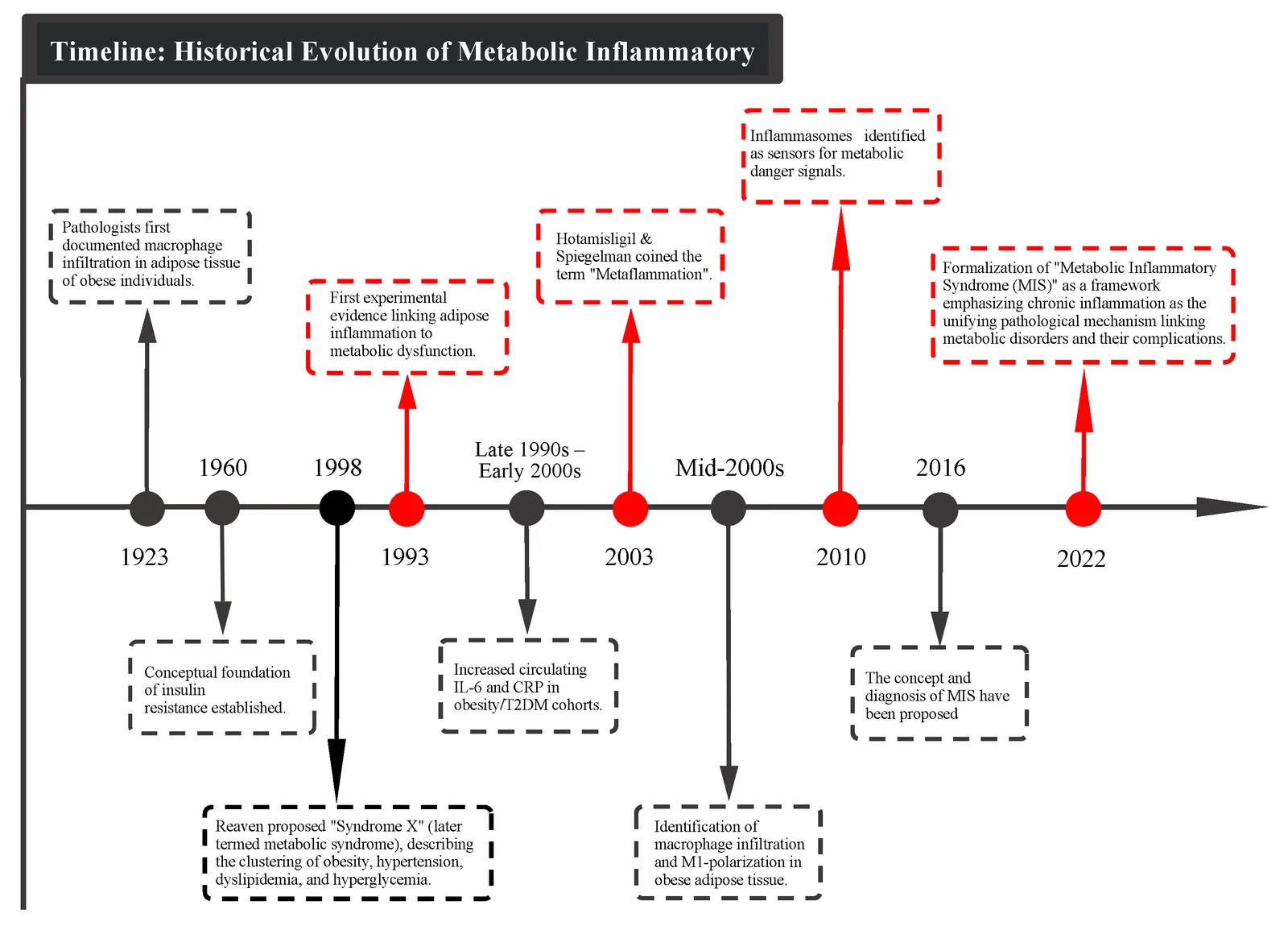
Timeline: Historical Evolution of MIS.

In 1998, the World Health Organization (WHO) formally defined metabolic syndrome, emphasizing insulin resistance as its core but not specifying the role of inflammation [20]. In the early 21st century, researchers are gradually discovered the critical role of chronic low-grade inflammation in metabolic diseases. The concept of MIS was first introduced by Hotamisligil in 2006 [21]. He suggested that nutrients and excess metabolism can act through toll-like receptors (TLRs) to trigger a chronic low-grade inflammatory response that leads to the development of metabolic diseases. In 2011, American experts in metabolic inflammation research also proposed that T2DM is a metabolic inflammatory disease [22]. In 2016, Renming Hu proposed the concept and diagnosis of MIS (known as Metaflammation) based on the association of atherosclerosis (AS), T2DM, NAFLD and obesity with metabolic inflammation [23].

**Table 1:** Differences Between MIS and Traditional MS.

Features	Metabolic Syndrome (MS)	Metabolic Inflammatory Syndrome (MIS)
Conceptual Core	MS is a phenotypic and clinical diagnosis. It identifies individuals at risk based on measurable cardiometabolic parameters.	MIS is a pathophysiological and mechanistic construction. It posits that chronic, low-grade inflammation, driven by nutrient excess and metabolic dysfunction in immune and metabolic cells, is the central unifying driver.
Diagnosis	The diagnosis of MS relies on meeting a threshold number of clinical criteria (e.g., ATP III criteria).	MIS diagnosis, while still evolving, aims to incorporate quantifiable biomarkers of inflammation (hs-CRP, IL-1β, IL-6, or omics-based signatures) alongside traditional metabolic criteria, allowing for earlier identification of at-risk individuals.
Clinical Implication	MS describes what the clinical risk factors are.	MIS explains why and how they cluster and progress.
Therapeutic Targeting	The management of MS has traditionally focused on treating each component (statins for dyslipidemia, antihypertensives for blood pressure).	The concept of MIS directs therapeutic strategies toward breaking the core inflammation-metabolism cycle itself. This suggests a rationale for targeting novel inflammatory pathways (IL-1β inhibition) or upstream drivers (gut microbiota modulation, cellular metabolic reprogramming) as foundational interventions.

### Metabolic Pathway Remodeling in Metabolic Inflammatory Syndrome

A growing body of evidence suggests that metabolic reprogramming serves as a fundamental pathogenic driver in MIS, orchestrating crosstalk between metabolic dysregulation and chronic inflammation [24]. This remodeling of cellular bioenergetic pathways, including glucose metabolism, lipid metabolism and amino acid metabolism, transforms metabolites into signaling molecules that activate immune cascades, which activate the inflammatory response [25] (Table 2).

**Table 2:** Metabolic disorders and associated inflammation in MIS.

Types	Main metabolites	Inflammatory factors/pathways
Glucose Metabolism	AGEs, Pyruvate, Lactate	NF-KB, IL-6, TNF-α,
Lipid Metabolism	LDL, sdLDL,TG, FFA	TNF-α, IL-1β, IL-6, TLR4, JNK/IKK, NF-KB
Insulin Metabolism	Citric acid, Succinic acid, Fatty acids, Pyruvic acid, AGEs, SCFAs, BCAA, Bile acids, Indole sulfuric acid, Trimethylamine oxide, Arachidonic acid	IL-1B, IL-6, TNF-α, ROS, MCP-1, IL-10, TLR4/NF-KB, VEGF, COX-2, PGI2, TXA2, NOX2

### Glucose Metabolism

The changes in glucose metabolism in MIS are a chain process initiated by inflammation of adipose tissue, which affects the liver, muscles, and pancreas through inflammatory factors and free fatty acids, ultimately leading to systemic insulin resistance and breakdown of blood glucose homeostasis (Fig. 2). It is characterized by enhanced aerobic glycolysis in immune cells and metabolic tissues (adipose, liver), coupled with suppressed mitochondrial oxidative phosphorylation [26, 27]. Mechanistically, upregulation of glycolytic enzymes hexokinase 2 and pyruvate kinase isozyme type-M2 induces lactate accumulation, activating hypoxia inducible factor-1α and increasing pro-inflammatory cytokines IL-1β and TNF-α. Shunting of glucose to the pentose phosphate pathway (PPP) causes NADPH overproduction, triggering ROS bursts that activate the NLRP3 inflammasome. Additionally, impaired Glucose Transporter 4 internalization in insulin-target cells (myocytes, hepatocytes) reduces glucose uptake, leading to hyperglycemia-induced accumulation of advanced glycation end products (AGEs) and subsequent RAGE/NF-κB signaling upregulation [28-30].

It is important that high glucose concentrations enhance the binding of NF-κB to DNA, reduce the expression of IκB kinase (IKK), and promote its degradation via the ubiquitin pathway [31]. Moreover, high glucose can increase the levels of pro-inflammatory transcription factors AP-1 and Egr-1. Meanwhile, high fat can directly activate protein kinase C (PKC) through TLR-4, inducing the activation of two downstream inflammatory pathways, IKK and JNK, causing insulin resistance and disrupting the balance of energy metabolism in the organism [32].

**Figure 2.**
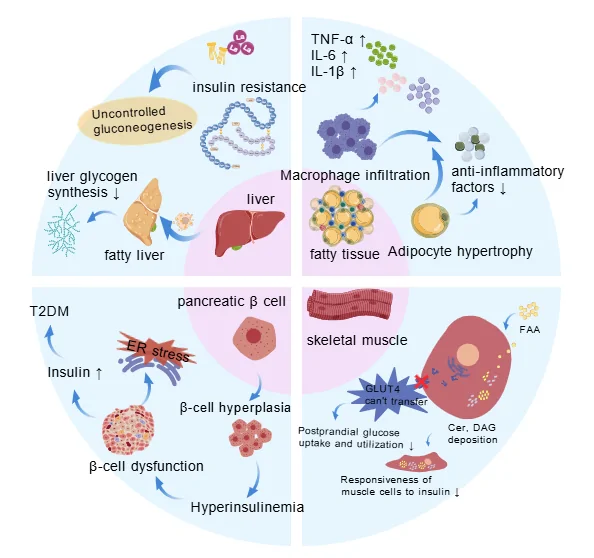
**Altered glucose metabolism in MIS**. In a state of metabolic inflammation, key metabolic tissues in the body (liver, fatty tissue, pancreatic β cell and skeletal muscle) block normal insulin signaling, leading to obstacles in various stages of glucose metabolism. Liver: Hepatic insulin resistance leads to persistent, excessive conversion of non-glucose substances into glucose, resulting in uncontrolled gluconeogenesis. Excess free fatty acid (FFA) flood from the bloodstream and accumulate in the liver, forming non-alcoholic fatty liver disease. This impairs glycogen synthesis, reducing the liver's capacity to take up glucose and convert it into glycogen for storage; Fatty tissue: Adipocyte hypertrophy and hypoxia recruit large numbers of immune cells (primarily M1 macrophages) to infiltrate adipose tissue. Hypertrophied adipocytes and infiltrating immune cells secrete substantial amounts of pro-inflammatory cytokines such as TNF-α, IL-6, and IL-1β, while simultaneously reducing the secretion of anti-inflammatory factors; Pancreatic β cell: Compensatory hypertrophy of pancreatic β-cells leads to excessive insulin secretion, manifesting as hyperinsulinemia. Prolonged exposure to glucotoxicity and lipotoxicity induces endoplasmic reticulum stress (ERS) and oxidative stress in β-cells. Sustained stress leads to β-cell dysfunction. Insulin secretion fails to compensate for insulin resistance, resulting in the development of T2DM; Skeletal muscle: Elevated levels of FFAs in the bloodstream enter muscle cells and accumulate as lipid intermediates (ceramides, diacylglycerols), disrupting insulin signaling. Insulin fails to effectively promote the transport of GLUT4 transporters to the cell membrane, resulting in reduced glucose uptake and utilization by muscle cells. This figure was created using the Generic Diagramming Platform (GDP) [146].

### Lipid Metabolism

Liver lipid metabolism is regulated by the intake and output of fatty acids, de novo fatty acid synthesis, and fatty acid utilization through β-oxidation [33]. Under healthy conditions, excess energy is stored as triglycerides (TG) in subcutaneous adipose tissue (SAT). However, in the metabolic inflammatory state, the storage function of adipose tissue becomes dysfunctional, leading to an overflow of FFA into non-adipose organs (liver, muscles, pancreas and blood vessels), causing lipotoxicity, which further exacerbates inflammation and insulin resistance [34].

Excess energy leads to excessive enlargement of fat cells, especially visceral adipose tissue (VAT) cells (Fig. 3). Enlarged VAT cells become insensitive to the anti-lipolytic effects of insulin. The hallmark of lipid metabolism reprogramming is a lipotoxic microenvironment driven by hyperactivated de novo lipogenesis (DNL) and inhibited fatty acid oxidation (FAO) [35, 36]. SREBP-1c-mediated DNL upregulation elevates saturated fatty acid (SFA) levels, inducing ERS and activating the IRE1α/XBP1 pathway, which promotes insulin resistance via JNK phosphorylation. Suppression of CPT1A, a key FAO enzyme, causes long-chain acyl-CoA accumulation, activating PKC and impairing insulin signaling through IRS serine phosphorylation. Concurrently, increased ceramide synthesis induces mitochondrial fission, releasing mtDNA that activates the cGAS-STING pathway and IFN-β production [37-39].

**Figure 3.**
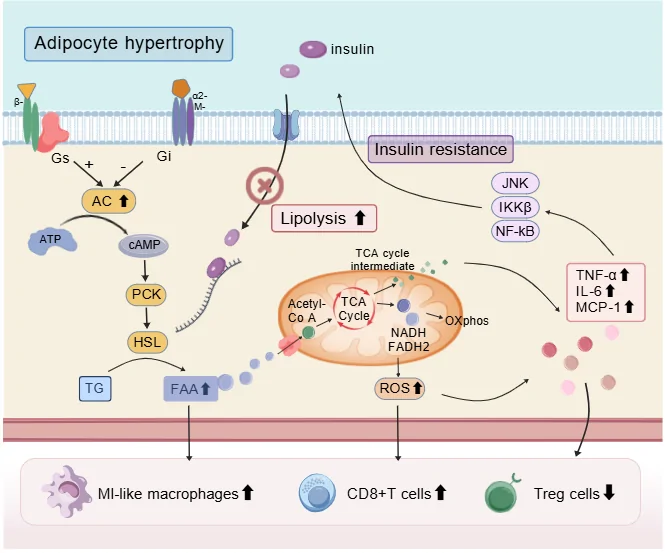
**Altered lipid metabolism in MIS**. Enhanced lipolysis: Energy excess leads to excessive hypertrophy of adipocytes. Adipocytes develop insulin resistance, diminishing their ability to inhibit HSL. The basal lipolysis rate increases, resulting in the sustained release of large amounts of free fatty acids into the bloodstream. Insulin Resistance: Inflammatory signals (TNF-α and IL-6) inhibit insulin signaling by activating JNK/IKKβ and NF-κB pathways, causing adipose tissue itself to become insulin-resistant. Oxidative stress: Elevated FFA concentrations activate acyl-CoA, which enters mitochondria via the carnitine palmitoyltransferase system for β-oxidation. This generates large amounts of NADH and FADH_2_, leading to massive ROS production. ROS acts as potent inflammatory signals, activating pathways like NF-κB. Immune Cell Infiltration: Stressed hypertrophic adipocytes secrete pro-inflammatory cytokines, recruiting circulating immune cells into adipose tissue. AC: adenylate cyclase; PCK: phosphoenolpyruvate carboxy kinase; HSL: hormone-sensitive lipase; TG: Triglyceride; FFA: free fatty acid; ROS: reactive oxygen species. This figure was created using the GDP.

### Amino Acid Metabolism

Under metabolic inflammatory conditions, protein turnover and amino acid catabolism become disrupted, leading to abnormal accumulation of specific amino acids in the blood and tissues. These accumulated amino acids and their metabolic intermediates can exacerbate insulin resistance and chronic inflammation [40, 41]. Amino acid remodeling centers on impaired branched-chain amino acid (BCAA) catabolism and activation of the tryptophan-kynurenine (Trp-Kyn) axis [42]. BCAAs are elevated in obesity, activate the mTOR pathway, inhibit insulin signaling and promote inflammation [43]. BCAA (leucine, isoleucine) accumulation in adipose/liver tissues chronically activates mTORC1, leading to S6K1-mediated IRS-1 phosphorylation and reduced insulin sensitivity [44]. Inflammation-induced IDO1 (indoleamine 2,3-dioxygenase) expression converts tryptophan to kynurenine, activating the aryl hydrocarbon receptor (AhR) to drive Treg differentiation while suppressing Th17 cells [45].

### Metabolic Reprogramming Drives Immune Cell Polarization and Inflammatory Storms

The core characteristic of MIS lies in the significant pro-inflammatory alterations induced in core immune cells within its typical hyperglycemic, hyperlipidemic and hypoxic microenvironment. Among these changes, macrophage polarization and functional alterations are essential factors of chronic inflammation and metabolic dysregulation. Analyzing the primary and secondary drivers of MIS progression will help prioritize potential therapeutic targets.

### The Core Drivers: Immune Cell Polarization and Dysfunction Induced by Metabolic Reprogramming

Macrophages are generally classified into pro-inflammatory (M1 macrophages) and anti-inflammatory (M2 macrophages) types, maintaining a relative equilibrium under physiological conditions [46]. Under metabolic stress, adipocytes produce large quantities of cytokines (CCL-2, MIPs, IL-6, IL-1β, TNF-α) [47], which recruit monocytes to adipose tissue and promote their differentiation into M1 macrophages. Through the action of macrophage chemoattractant protein-1 (MCP-1), a positive feedback loop is established, recruiting additional M1 macrophages to infiltrate the tissue [48, 49]. In obesity, macrophages can expand to constitute over 40% of all immune cells. Their polarization and dysregulated inflammatory phenotype induce systemic complications (such as insulin resistance, hyperglycemia and dyslipidemia), increasing the risk of metabolic diseases [50].

Polarized M1 macrophages are characterized by HIF-1α and mTORC1 signaling-driven hyperactivation of glycolysis, rapidly generating energy and producing large quantities of pro-inflammatory cytokines (such as TNF-α, IL-1β, and IL-6) [51, 52]. Concurrently, mitochondrial dysfunction manifests as impaired oxidative phosphorylation and disrupted tricarboxylic acid (TCA) cycle activity, leading to succinate accumulation [53]. Succinate not only stabilizes HIF-1α but also promotes ROS generation and activates the NLRP3 inflammasome. Fatty acid metabolism is also severely disrupted. Increased SFA uptake via CD36 activates the TLR4 inflammatory pathway, while suppressed FAO leads to intracellular lipid accumulation and lipotoxicity [54, 55]. These changes establish M1 macrophages as primary drivers of inflammation, insulin resistance, and fibrosis in metabolic tissues like adipose and liver.

The immunopathology of MIS also includes significant perturbations in T lymphocyte subsets [56]. Under overactive mTORC1 signaling, effector T cells such as Th1 and Th17 become highly dependent on glycolysis for proliferation, secreting factors like IFN-γ and IL-17 that directly disrupt insulin signaling [57]. Concurrently, Treg functionality is impaired due to disruptions in FAO and oxidative phosphorylation, resulting from AMPK inhibition. Infiltration of these Treg into metabolic tissues facilitates synergistic interactions with macrophages, thereby exacerbating local and systemic inflammation [58, 59].

Neutrophils exhibit enhanced basal glycolysis, resulting in excessive ROS production. Under hyperglycemic conditions, they form pro-damage neutrophil extracellular traps (NETs), acting as early responders that cause direct oxidative tissue damage and recruit additional inflammatory cells [60, 61].

Dendritic cells also undergo metabolic reprogramming in hyperlipidemia, marked by enhanced glycolysis and lipid accumulation. This alters their function, leading to abnormal activation of Treg responses, thereby enhancing innate and adaptive immune responses [62, 63].

### Key amplifiers: Specific inflammatory pathways triggered by metabolic abnormalities

Inflammasome activation and specific cytokine signaling (NLRP3/IL-1β, TNF-α, JNK/NF-κB) constitute key effector pathways directly activated and significantly amplified by core metabolic reprogramming. Dasu et al. found a significant increase in the secretion of pro-inflammatory factors (MCP-1, IL-1β, IL-6, IL-8) by inducing macrophage infiltration in human pancreatic islets through hyperglycemia and hyperlipidemia [64]. Inflammatory cytokines and FFA could interfere with the insulin signaling pathway by activating the NF-κB and JNK signaling pathways in macrophages, mediating obesity-related insulin resistance [65]. L-1β produced by macrophages can induce islet cell death by upregulating pro-apoptotic receptors on the islet cell surface [66]. Moreover, NLRP3 inflammasome has been considered as a link between lipid metabolism and inflammation because crystalline cholesterol and oxidized low-density lipoprotein (oxLDL) (two abundant components in atherosclerotic plaques) activate NLRP3 inflammasome [67]. Lipotoxicity promotes insulin resistance through pathways such as IRE1α/JNK [68]. The prolonged activation of JNK could be alleviated by NF-κB-mediated upregulation of anti-apoptotic genes, such as Bcl-xl and cFLIP, which may represent a potential therapeutic strategy [69].

### Downstream Effects and Modulators: Specific Pathological Alterations Observed in Organs or Tissues

Insulin resistance, tissue fibrosis, and organ dysfunction (in the pancreas, liver, adipose tissue, blood vessels) represent the integrated manifestation and ultimate outcome of core drivers and key amplified signals within specific organs. Insulin resistance leads to increased hepatic glycogenesis, reduced muscle glucose uptake, decreased lipolysis, and enhanced hepatic lipid synthesis, resulting in elevated blood glucose levels and increased circulating FFA concentrations [70]. To protect the body from the effects of elevated FFA levels, adipose tissue undergoes hypertrophy and hyperplasia. Impaired FAO, combined with adipose tissue deposition, contributes to the development of fatty liver [71].

Other factors, such as gut microbiota metabolites and iron metabolism disorders, also modulate the intensity and progression rate of core drivers rather than directly causing MIS. For instance, when macrophages were polarized, iron transfer between macrophages and adipocytes in co-culture was accelerated, with a net increase in iron accumulation in adipocytes [72]. Adipocyte iron concentration is associated with systemic metabolic health because adipocyte-specific overload due to iron transporter protein deficiency leads to glucose intolerance, and lowering adipocyte iron through transferrin receptor deficiency prevents obesity-related metabolic dysfunction [73, 74]. In contrast, low macrophage iron content was associated with an M2-like phenotype and improved glucose tolerance [75]. These factors determine individual susceptibility to MIS and disease phenotype heterogeneity, making them essential considerations for personalized medicine.

### The Gut Microbiota Plays an Important Role in The Metabolism-Inflammation Axis

The gut microbiota communities not only maintain metabolic homeostasis but actively drive MIS progression via a sophisticated bidirectional communication network between the gastrointestinal tract and the liver [76, 77]. The liver regulates the composition and function of the gut microbiota through bile acids (BAs) and inflammatory mediators. In turn, gut microbiota transport metabolites and signaling molecules through the portal venous circulation, which modulate hepatic bile acid synthesis, glucose and lipid metabolism, and inflammatory responses [78]. Disruption of this equilibrium manifests as intestinal dysbiosis, impaired gut barrier integrity, and amplified hepatic inflammation [79].

BAs, as key molecular mediators, coordinate interactions between the microbiota and the host through dual regulatory functions [80, 81]. Exogenous administration of glycine-β-muricholic acid reduces the *Firmicutes*/*Bacteroidetes* ratio, decreases substrates for hepatic DNL, and consequently ameliorates obesity-associated metabolic dysfunction [82]. The supplementation of aromatic hydrocarbon receptor agonists or strains with high aromatic hydrocarbon receptor ligand production capacity can effectively improve diet-induced and genetic metabolic disorders, particularly glucose metabolism disorders and hepatic steatosis, by enhancing intestinal barrier function and promoting the secretion of the intestinal hormone glucagon-like peptide-1 (GLP-1) [83]. Significantly, BA derivatives such as tauroursodeoxycholic acid enhance insulin sensitivity in hepatic and muscular tissues by mitigating ERS, highlighting the therapeutic relevance of BA signaling pathways in metabolic inflammation [84]. Conversely, the gut microbiota precisely regulates BAs metabolism through farnesoid X receptor signaling [85].

In addition, exogenous pathogen-associated molecular patterns (such as LPS and SCFAs) can also cause chronic low-grade inflammation [86, 87]. SCFAs can regulate gut peptide secretion by recognizing receptors on intestinal cell surfaces, exerting positive effects on reducing food intake and improving glucose metabolism [88]. LPS may promote the development of MIS by triggering systemic chronic low-grade inflammation [89]. These findings highlight the gut microbiome's multifaceted mechanisms for influencing host metabolism and inflammatory states through its metabolites.

The heterogeneity of the gut microbiome is a core challenge in understanding its complex association with MIS. This heterogeneity is primarily reflected in species composition, functional gene profile, and metabolic products, and is closely associated with factors such as host diet, genetic background, age, and medication history [90, 91]. Numerous clinical studies have demonstrated that the distinct clinical phenotypes of MIS (obesity, T2DM, NAFLD, and CVD) do not correspond to a single dysbiosis pattern. Instead, they exhibit highly specific microbial community characteristics (Table 3). At the microbial community diversity level, patients with MIS generally exhibit a decrease in microbial α-diversity (species richness and evenness). In NAFLD/MAFLD, there is often an enrichment of Enterobacteriaceae and a reduction in *Bifidobacterium*, accompanied by abnormalities in bile acid metabolism and hepatic lipid metabolism. In T2DM, notable changes in microbiota such as *Clostridium*, *Desulfovibrio*, *Escherichia coli*, and *Roseburia* are observed, which are linked to disruptions in glucose metabolism. Among AS and CVD, an increase in *Bilophila wadsworthia, Ruminococcus gnavus*, and *Streptococcus* are commonly observed, alongside a decrease in beneficial bacteria such as *Faecalibacterium prausnitzii* and *Roseburia*. At the functional level, all MIS subtypes exhibit disturbances in core metabolic pathways to varying degrees. A common feature across all subtypes is the reduced capacity for SCFA biosynthesis, particularly a decline in the production of butyrate and propionate, which is closely associated with intestinal barrier impairment and systemic inflammation. By harnessing the heterogeneity of the gut microbiome, clinicians could stratify MIS risk, optimize personalized prevention and treatment approaches, and potentially slow or reverse the progression of this complex metabolic-inflammatory disorder.

**Table 3:** Comprehensive profiling of gut microbiota in MIS patient cohorts.

Different MIS phenotypes	Characteristics of gut microbiota		Altered metabolic function	Ref.
	Increased microbiota in the patient	Reduced microbiota in the patients		
NAFLD/MAFLD	Escherichia, S higella, *Lachnospiraceae*, *Prevotella*	*Eubacterium*, *Bacteroides*, *Christensenel laceae, Ruminococcus*	Bile acid metabolism, Liver Fat Metabolism	[131]
Metabolic syndrome	*Klebsiella, Lactobacillus, Streptococcus*	*Akkermansia muciniphila*	SCFA, Bile acid metabolism, Insulin resistance, Glucose metabolism, Liver Fat Metabolism	[132]
T2DM	*Clostridium, Desulfovibrio, Escherichia,Escherichia coli, Lachnospiraceae, Roseburia, Roseburia inulinivorans*		Bile acid metabolism, Liver Fat Metabolism	[133]
Metabolic syndrome	*Prevotella*	*Bacteroides*	Altered inflammatory pathways, Insulin resistance, Glucose metabolism, Fat Metabolism	[134]
T2DM, Obesity	*Lactobacillus*	*Klebsiella*	Glucose metabolism, Liver fat metabolism	[135]
T2DM, Obesity, AS		*Akkermansia muciniphila*	Altered inflammatory pathways, Insulin resistance, Glucose metabolism	[136, 137]
AS	*Prevotella*	*Bifidobacterium*		[138]
T2DM, Obesity, AS	*Lachnospiraceae, Ruminococcus, Ruminococcus gnavus, Prevotella, Roseburia*,	*Bacteroides, Bilophila, Faecalibacterium prausnitzii, Streptococcus*	TMAO, Altered inflammatory pathways, Insulin resistance, Bile acid metabolism	[139]
T2DM	*Eubacterium, Ruminococcus*		SCFA, Bile acid metabolism, Glucose metabolism	[140]
NAFLD/MAFLD	*Enterobacteriaceae*	*Bifidobacterium, Enterobacteriaceae*	Bile acid metabolism, Liver fat metabolism	[141]
NAFLD/MAFLD		*Bacteroides, Bacteroides stercoris, Parabacteroides, Clostridium*	Altered inflammatory pathways, Insulin resistance, Liver fat metabolism, BCAA Metabolism	[142, 143]
T2DM, NAFLD, AS/CVD	*Lactobacillus, Parabacteroides, Ruminococcaceae*	*Akkermansia muciniphila, Bacteroides, Blautia, Desulfovibrio, Lachnospiraceae*	SCFA, Bile acid metabolism, TMAO, Liver fat metabolism	[144]
Obesity, AS/CVD	*Alistipes, Clostridium, Eubacterium, Eubacterium rectale, Ruminococcus*	*Bacteroides, Bacteroides vulgatus*	Bile acid metabolism, Altered inflammatory pathways, Glucose metabolism, Liver fat metabolism	[145]

Notably, the composition and function of the gut microbiome exhibit distinct age-related dynamic changes. Older populations commonly display reduced microbial diversity, decreased SCFA-producing bacteria (*Faecalibacterium prausnitzii* and *Roseburia*), and increased pro-inflammatory microbiota, which are closely associated with the high prevalence of MIS [17, 18]. Particularly noteworthy is that the origins of MIS risk can be traced back to early life. During youth, prolonged high-fat/high-sugar diets, antibiotic exposure, and chronic stress may disrupt the gut microbiota, leading to endotoxemia, endothelial dysfunction, and subclinical atherosclerosis, significantly accelerating the onset of CVD [92]. Therefore, implementing microbiota-targeted early interventions in young populations holds significant preventive value.

Therefore, interventions targeting the microbiome, such as dietary modifications (Mediterranean diet, high-fiber diet, time-restricted eating), prebiotics (human milk oligosaccharides), probiotics (*A.muciniphila, Bifidobacterium*, and *Lactobacillus*), and specific nutritional supplements (curcumin and omega-3), can partially reverse age-related dysbiosis and the progression of MIS by modulating microbiota-host interaction pathways [93, 94]. Importantly, the high degree of heterogeneity in the gut microbiome significantly influences the design and efficacy of these therapeutic strategies. For instance, while prebiotics consistently increase the abundance of beneficial bacteria such as *Bifidobacterium*, their effects on glycemic parameters vary markedly across studies. Some trials report no significant reduction in HbA1c [95], whereas others demonstrate beneficial metabolic outcomes [96]. These differential responses may be attributed to variations in baseline microbial architecture and intervention duration. Similarly, dietary interventions such as the Mediterranean diet have been shown to promote *Prevotella* and *A. muciniphila*, accompanied by improvements in hepatic steatosis and insulin resistance. However, the magnitude of these benefits is influenced by baseline dietary patterns, regional food matrices, and host genetic factors. Therefore, personalized microbiome therapies tailored to an individual's unique microbial profile can yield more effective MIS prevention and treatment strategies, ultimately offering new perspectives for individualized health management.

Although alterations in microbial metabolites caused by ecological imbalance (such as decreased SCFAs and elevated LPS) may contribute to the heterogeneous clinical manifestations of MIS, we cannot overlook the crucial role of other host-derived factors in the disease pathogenesis. Genetic susceptibility (polymorphisms in genes involved in insulin signaling pathways), epigenetic modifications (DNA methylation and histone modifications regulating metabolism-related genes), and organ-specific metabolic dysfunctions (hepatic lipid accumulation, abnormal adipose tissue secretion profiles, and skeletal muscle insulin resistance) profoundly influence the onset and progression of MIS [97, 98]. Relying solely on gut microbiome data risks overlooking these critical host factors. Therefore, there is a clear need for developing predictive models that integrate gut microbiome data with other clinical and biochemical parameters to enable quantitative risk assessment and early identification of individuals predisposed to MIS.

### Innovative therapeutic targets: From Mechanisms to Precision Interventions

The pathological complexity of metabolic syndrome has driven the exploration of multi-target therapeutic strategies (Table 4). Current efforts focus on three core directions: inhibiting glucose metabolism, modulating insulin signaling, and reprogramming lipid metabolism. The overarching goal is to enhance insulin sensitivity and reshape the inflammatory microenvironment by targeting key metabolic enzymes.

Beyond traditional dietary interventions, GLP-1 has emerged as a powerful therapeutic agent for comprehensive metabolic risk reduction, demonstrating significant efficacy in obesity, T2DM, and MASLD [99, 100]. It regulates glucose levels, lipid metabolism, and various physiological functions by enhancing insulin secretion, reducing pancreatic β-cell apoptosis, suppressing glucagon production and hepatic glucose output, slowing gastric emptying, and decreasing appetite. Their mechanism extends to include direct and indirect anti-inflammatory effects, crucial for disrupting the sub-inflammatory cycle of MIS, such as modulating macrophage polarization via STAT3 [101].

Similarly, metformin is widely used as a first-line drug for T2DM. It increases insulin levels by reducing hepatic glucose output and enhancing glucose sensitivity [102]. Recent clinical evidence confirms its benefits on oxidative stress and inflammation in MetS patients [103], with studies showing it dampens monocyte-to-macrophage differentiation and inhibits JNK/NF-κB pathways [104, 105]. The key barrier for these repurposed drugs is not efficacy but optimizing their use in specific MIS patient subgroups.

Another important approach involves controlling key inflammatory regulators (such as the JNK and IKK pathways) rather than targeting individual cytokines alone. Preclinical studies indicate that salicylate (an IKK inhibitor) improves glucose metabolism in obese mice and diabetic patients [106]. Inhibiting other inflammatory kinases may yield similar benefits [107]. Targeting JNK via peptides or small molecules enhances insulin action in mouse models [108]. However, the clinical translation of these pathway inhibitors faces significant hurdles. Developing effective oral small-molecule inhibitors with sufficient JNK isoform selectivity has proven challenging. More critically, safety concerns regarding systemic immunosuppression pose a primary obstacle, as evidenced by the narrow therapeutic window for high-dose salicylates. Currently, these inhibitors remain primarily in the preclinical stage, with ongoing research focused on enhancing tissue specificity and safety profiles.

**Table 4:** Potential therapeutic targets of MIS.

Treatment direction	Targets	Category	Mechanism of action
Inhibition of glucose metabolic pathway	Glucagon receptor	GPCR	Antagonists inhibit gluconeogenesis and glycogenolysis.
	Glycogen phosphorylase	enzyme	Prevent glycogen degradation to glucose.
	Fructose 1,6-bisphosphate	enzyme	Blocking gluconeogenesis
	Glucose-6-phosphatase	enzyme	Inhibit gluconeogenesis and glycogen decomposition
Regulating insulin related pathways	GLP-1 receptor	GPCR	Agonists promote insulin secretion, inhibit glucagon cord and delay gastric emptying
	DP-IV (DPP-4)	enzyme	Inhibit GLP-1 decomposition and enhance endogenous effect
	Exendin-4	peptide	Direct activation of GLP-1 can inhibit appetite and regenerate B cells
	PTP-1B	Phosphatases	Deactivation of insulin receptor and inhibition of insulin receptor can improve insulin sensitivity
	SHIP-2	Phosphatases	Dephosphorylate PIP3 and inhibit PI3K pathway
	GSK-3	kinase	Inhibit glycogen synthase and glucose metabolism
	IKK	kinase	It is involved in NF KB activation and is associated with insulin resistance
	PKC	kinase	FAA induced insulin resistance
Regulating lipid metabolism pathway	AMPK	enzyme	Activate fat oxidation, inhibit fat synthesis and enhance glucose uptake
	ACC	enzyme	Increased fat oxidation and reduced body fat
	Acrp30	kinase	Enhance insulin sensitivity and reduce tissue lipid toxicity
	PPAR-γ/α	Nuclear receptor	Regulation of lipid metabolism and insulin sensitivity
	11β-HSD1	enzyme	Local activation of glucocorticoids is associated with insulin resistance
Other potential targets	MC4R	PGCR	Control central appetite regulation and energy consumption
	Fatty acid synthase	enzyme	Central inhibition can reduce appetite and fat synthesis
	Resistin	cytokine	It is related to insulin resistance and may be a therapeutic target
	TNF-α	cytokine	Proinflammatory factor, regulating its expression can improve insulin sensitivity

Emerging targets, such as mitochondrial dysfunction and ERS, show great promise, as correcting these defects can suppress central inflammatory drivers of MIS [109]. Despite compelling preclinical data, these targets remain in the early stages of translational development. Major obstacles include the lack of specific drugs applicable to humans and challenges in non-invasively monitoring binding to relevant tissue targets during clinical trials.

### Diagnostic Biomarkers

Alongside the current early warning indicators, there are additional indicators that have not been extensively utilized in clinical settings (Table 5). These need further optimization within the testing system to enhance the early screening and intervention for MIS.

Hypersensitive C-reactive protein (hs-CRP), as a chronic inflammatory marker in diabetic patients, serves as a sensitive indicator of vascular inflammation [110]. The mechanisms by which hs-CRP contributes to AS include mediating various chemokines and adhesion molecules involved in its development, activating the complement system—which plays a role in AS formation—and interacting with other factors, such as inflammatory mediators like IFN-γ and LPS, to promote AS [111, 112]. It exhibits high sensitivity, effectively reflecting systemic low-grade inflammatory states. However, its specificity is relatively low. It has been used for routine cardiovascular risk assessment due to its ease of testing and high cost-effectiveness.

Serum lipoprotein-associated phospholipase A2 (Lp-PLA2) hydrolyzes phospholipids, releasing inflammatory mediators such as lysophosphatidic acid and oxidized free fatty acids. These factors attract more monocytes to the lesion site, leading to the formation of foam cells when they combine with oxidized low-density lipoprotein (LDL). Lp-PLA2 plays a significant role in the formation, development, and rupture of unstable plaques, which can ultimately result in acute cardiovascular and cerebrovascular events [113, 114]. A retrospective cohort study revealed a significant interaction between Lp-PLA2 activity and diabetes control status, identifying individuals with both high Lp-PLA2 activity and poorly controlled diabetes as a high-risk subgroup who derived a 33% reduction in major cardiovascular events from Lp-PLA2 inhibition [115]. Compared to hsCRP, Lp-PLA2 exhibits higher specificity for vascular inflammation and has received regulatory approval for cardiovascular risk stratification, making it an effective tool for assessing vasculitis-specific inflammation.

**Table 5:** Potential Diagnostic Biomarkers for Metabolic Inflammatory Syndrome.

Biomarker	Key Function	Sensitivity	Specificity
Lp-PLA2	Independent predictor of cardiovascular events	Moderately High (70-80%)	Moderate
MPO	Key warning indicator for acute cardiovascular events	High (esp. acute phase >90%)	Low
hs-CRP	Systemic low-grade inflammation	High (>80%)	Low
sST2	Cardiac fibrosis, remodeling, dysfunction; Potential for early warning	High (>80%)	High (>80%)
Complement C3	Innate immunity-metabolic disorder link	Relatively High (>70%)	Moderate
TNF-α	Pro-inflammatory cytokine; insulin resistance	High (0.1 pg/ml)	Low
LXR/PPAR	Regulate metabolism/inflammation	No relevant data available	No relevant data available

Myeloperoxidase (MPO) is released in response to inflammatory reactions within the body, which plays a significant role in the mechanisms of AS plaques by catalyzing reactions that consume nitric oxide (NO) and inhibit its biological activity [116]. It mediates and enhances the nitrosation of NO, further disrupting vascular endothelial function [117, 118]. Additionally, MPO works alongside hydrogen peroxide and chloride ions to promote cholesterol accumulation and foam cell formation, contributing to plaque development. The advantage of MPO lies in its high sensitivity, which can exceed 90% during the acute phase of acute coronary syndrome. However, it can be elevated in various inflammatory conditions, resulting in relatively low specificity [119, 120]. Currently, MPO is primarily used for risk stratification and prognosis assessment in patients with acute chest pain and is not employed as a routine screening marker.

Soluble growth stimulation expressed gene 2 (sST2) is a potential target for drug action in conditions related to inflammation, oxidative stress, and fibrosis. In a cross-sectional study involving fifty-eight patients with metabolic syndrome, serum sST2 levels were found to be significantly higher than the normal mean values (15.7-18.9 ng/ml). These elevated levels were strongly correlated with oxidative stress, inflammation, and various echocardiographic parameters [121]. Thus, sST2 may serve as an early indicator of cardiac pathological remodeling and dysfunction in patients with metabolic syndrome. However, its sensitivity and specificity as an early biomarker for cardiac dysfunction in metabolic syndrome remain under investigation.

Complement 3 (C3) and Complement 4 (C4) are key biologically active proteins involved in the complement system, primarily synthesized by hepatocytes and macrophages. Levels of C3 and C4 are strongly associated with the diagnosis of metabolic syndrome [122, 123]. Among these, C3 demonstrates high sensitivity for diagnosing metabolic syndrome, but its specificity is moderate, as factors such as obesity and infection can also influence its concentration. Serving as a bridge linking innate immunity to metabolic disorders, it holds some value in risk identification but has not yet become an independent diagnostic criterion.

The combination and role of cytokines in MIS is a crucial yet understudied area. Some transcription factors, such as liver X receptor (LXR) and peroxisome proliferator-activated receptor (PPAR), critically regulate metabolic pathways [124, 125]. TNF-α acts as a pro-inflammatory cytokine that activates various signaling cascades and is a key inhibitor of insulin action. Preliminary research demonstrated that TNF-α expression was upregulated in adipose tissue of obese mice, thereby identifying the previously unrecognized association between obesity, diabetes, and chronic inflammation [126]. Additionally, TNF-α targets and modulates this component of insulin receptor signaling by inhibiting the serine phosphorylation of IRS-1 [127]. Increasing evidence suggests that activation of JNK, IKK, and PKC is central to mediating insulin resistance in response to a variety of stresses that occur in obesity and other insulin-resistant conditions [128, 129]. TNF-α currently serves primarily as a core molecular target for mechanism studies and drug development, with its clinical readiness still confined to the research phase. Similarly, transcription factors such as LXR and PPAR are currently utilized mainly as research tools and drug development targets and have not yet been established as clinical diagnostic biomarkers.

In summary, these biomarkers constitute a multi-tiered risk assessment system spanning systemic inflammation, vascular-specific lesions, and cardiac fibrosis. Lp-PLA2 and hs-CRP have demonstrated clear clinical utility, while MPO and sST2 play significant roles in specific prognostic evaluations. Complement components, inflammatory cytokines, and transcription factors, meanwhile, shed deeper insights into disease mechanisms and point toward future therapeutic directions. Future clinical practice may increasingly favor the combined use of biomarkers at different preparation levels to achieve more precise risk stratification and enable earlier intervention.

## Discussion

The metabolic adaptation of MIS is complex and dynamic. The causes of metabolic dysfunction include nutritional deficiency and excess, which can activate inflammatory responses through the stress pathway. Nutritional imbalance aggravates the disorder of the metabolic inflammation axis through stress signals. Lipid and cytokines stimulate the inflammatory cascade through JNK/IKK kinase and ERS, while inflammatory factors such as TNF-α can reverse and aggravate ERS and ROS accumulation, block insulin signal, and expand the scope of inflammation. This positive feedback mechanism is the key driver of the continuous deterioration of metabolism. Metabolic disorders related to MIS involve abnormalities in glucose metabolism, lipid metabolism, and amino acid metabolism. A common feature of these disorders is their interconnection through chronic low-grade inflammation. VAT plays a crucial role as a source of inflammatory signals. Hormones and cytokines secreted by adipocytes and macrophages, such as TNF-α and MCP-1, not only trigger local inflammation in adipose tissue but also contribute to systemic insulin resistance and overall systemic inflammation. This creates a vicious cycle between metabolism and inflammation.

The gut microbiota plays a central role in MIS, as it links diet, metabolism, and innate immune responses. Clinical research has shown that an increase in *Aspergillus* and a decrease in butyrate-producing bacteria in the gut of elderly individuals are associated with higher systemic levels of pro-inflammatory cytokines, such as IL-6 and IL-8 [130]. It is therefore recommended to monitor the dynamic changes in gut microbial profiles during the course of MIS development and in response to different treatment modalities to guide adaptive, microbiome-informed management strategies.

Current therapeutic strategies for MIS continue to evolve, yet significant limitations remain. A major challenge lies in translating the increasingly detailed mechanistic understanding of MIS into safe and effective clinical interventions. Most available treatments focus primarily on managing downstream symptoms (such as with insulin sensitizers, lipid-lowering agents, or anti-inflammatory drugs) rather than targeting upstream drivers (like specific microbial dysbiosis). Interventions aimed at modulating the microbiome, including prebiotics, probiotics, and dietary modifications, have shown promise but produce inconsistent results. This variability stems largely from substantial interindividual differences in baseline gut microbiota, genetic predispositions, and lifestyle factors.

Furthermore, MIS is characterized by dysregulation across multiple interconnected pathways. Current research focuses on the immunomodulatory roles of metabolites such as FFAs and LPS, as well as the mechanisms underlying macrophage-helper cell interactions. However, the heterogeneity and diversity of immune cell populations pose considerable challenges to developing single-target therapies. Monotherapies directed at single molecular targets (such as specific receptors or enzymes) frequently demonstrate limited clinical efficacy, as inhibiting one inflammatory mediator often fails to disrupt the self-sustaining cycle of metabolic and immune dysfunction.

Therefore, it is necessary to conduct large-scale, multidisciplinary research using multi-omics approaches to validate its clinical relevance and identify novel therapeutic targets. Comprehensive regulatory approaches targeting metabolic-immune networks hold promises for pioneering new pathways in personalized management of MIS. ﻿
